# 246. Particularities of Non-axial Osteoarticular Tuberculosis Among Adults

**DOI:** 10.1093/ofid/ofab466.448

**Published:** 2021-12-04

**Authors:** Fatma Hammami, Makram Koubaa, Amal Chakroun, Khaoula Rekik, Chakib Marrakchi, Fatma Smaoui, Mounir Ben Jemaa

**Affiliations:** Infectious Diseases Department, Hedi Chaker University Hospital, University of Sfax, Tunisia, Sfax, Sfax, Tunisia

## Abstract

**Background:**

Osteoarticular tuberculosis (TB) represents 1% to 3% of all TB cases, among which spondylodiscitis is the most common presentation of the disease. Non-axial TB is less frequent. We aimed to study the clinical, therapeutic and evolutionary features of non-axial osteoarticular TB.

**Methods:**

We conducted a retrospective study including all patients hospitalized in the infectious diseases department for non-axial osteoarticular TB between 1999 and 2019.

**Results:**

We encountered 51 cases, among which 26 cases were males (51%). The mean age was 41±20years. Ten patients were previously treated for TB (19.6%). The revealing symptoms were fever (70.5%), asthenia (68.6%), weight loss (60.7%) and night sweets (43.1%). Arthritis was noted in 20 cases (39.2%) represented by TB of the hip (10 cases), knee (4 cases), shoulder (4 cases) and elbow (2 cases). There were 12 cases of sacroiliac osteoarthritis (23.5%) and 6 cases of femur osteomyelitis (11.7%). Other affected sites included sternum (7.8%), toe (5.9%), tibia (5.9%), mandible (2%), clavicle (2%) and mastoid bone (2%). Multifocal TB was noted in 12 cases (23.5%). Pulmonary TB was associated to osteoarticular TB in 13.7% of cases. The mean duration of antitubercular therapy was 10±5months. Fixed dose combinations were prescribed in 17.6% of the cases. The disease evolution was favorable in 47 cases (92.1%). Relapse was noted in 3 cases (5.8%) and death in one case (2%).

**Conclusion:**

Non-axial osteoarticular TB was not a rare disease. Multiple sites might be involved which facilitate the diagnosis confirmation. Prolonged antitubercular therapy might be required.

**Disclosures:**

**All Authors**: No reported disclosures

